# DNA Heat Treatment for Improving qPCR Analysis of Human Adenovirus in Wastewater

**DOI:** 10.1007/s12560-017-9294-4

**Published:** 2017-04-10

**Authors:** Emily Rames, Anne Roiko, Helen Stratton, Joanne Macdonald

**Affiliations:** 1 Genecology Research Centre, School of Science and Engineering, University of the Sunshine Coast, 90 Sippy Downs Dr, Sippy Downs, QLD 4556 Australia; 2 School of Medicine and Menzies Health Institute Queensland, Griffith University, Nathan, Australia; 3 School of Natural Sciences, Griffith University, Nathan, Australia; 4 Division of Experimental Therapeutics, Department of Medicine, Columbia University, New York, USA

**Keywords:** Adenovirus, QPCR, Inhibitors, Health risk, Wastewater, DNA heat treatment

## Abstract

PCR inhibitory substances in complex sample matrices can cause false negatives or under-estimation of target concentration. This study assessed DNA heat treatment for reducing inhibition during qPCR analysis of human adenovirus (HAdV) in wastewater samples. Inhibition was reduced by heat treating DNA, where mean HAdV concentration was increased by 0.71 log_10_ GC/L (and up to 3.04 log_10_ GC/L in one case), and replicate variability and false negatives were reduced. DNA heat treatment should be further investigated for improving reliability of HAdV concentration estimates in water, which can support more accurate assessment of health risks associated with viral pathogen exposure.

**Electronic supplementary material:**

The online version of this article (doi:10.1007/s12560-017-9294-4) contains supplementary material, which is available to authorized users.

Waterborne viruses are associated with illnesses such as gastroenteritis, meningitis, encephalitis and respiratory infections, and can be present in high levels in municipal wastewater (Bridge et al. 2010; Cantalupo et al. 2011; Sinclair et al. 2009). Methods for assessing viral contamination of wastewater are important for determining efficiency of treatment processes and for assessing risks associated with viral exposure due to (1) discharge of effluent into recreational water or drinking water supplies, and (2) re-use of treated wastewater, such as for irrigation in agriculture, sports fields and food processing (Bartram et al. 2001; Mara et al. 2010). Human adenovirus (HAdV) is used extensively as an indicator of viral contamination of water (Bofill-Mas et al. 2013; Rames et al. 2016). The use of qPCR for estimating HAdV concentration in water has advantages including much faster time to results and lower cost, which may facilitate more routine monitoring of viral contamination (Botes et al. 2013; Symonds and Breitbart 2015).

QPCR analyses of viruses in water can, however, can be impacted by molecular inhibitors. For example, a large scale analysis of enteric viruses in different water types showed 34% of 3193 samples would have been false negatives or viral concentration under-estimated, if qPCR inhibition was not assessed (Gibson et al. 2012). Inhibitors are co-purified during concentration and recovery of viruses from water samples, and interfere with amplification by their interaction with DNA, DNA polymerase or other reaction components (Schrader et al. 2012; Wilson 1997). Various methods have been used to reduce effects of inhibitors in qPCR, such as additional purification methods (e.g., chloroform extraction of viral concentrates) and use of facilitators such as bovine serum albumin in the qPCR reaction (Kreader 1996; Rodríguez et al. 2012). However, problems with inhibition have not been completely resolved using these methods (Botes et al. 2013; Rodríguez et al. 2012).

This study assessed an additional means for reducing inhibition, by heat treating viral DNA from wastewater samples prior to qPCR. DNA from viral concentrates of waste stabilisation pond (WSP) inlet and outlet samples (*n* = 22), which were collected during a study of WSP effectiveness (Scheludchenko et al. 2016), were analysed in HAdV qPCR as described in Supplementary Methods, using primers/probe reported by Heim et al. (2003). Inhibition was initially assessed by spiking undiluted (1:1) DNA with 10^3^ gene copies (GC) of a control adenovirus 41 plasmid. Results indicated qPCR was inhibited in three of the four samples tested (Table S1), as the observed qPCR estimate was <60% of the expected value (spiked GC + endogenous sample GC). Inhibition was then further assessed using an alternative method, where sample DNA dilutions (without spiking) were tested, to dilute out effects of inhibitors. Comparison of HAdV concentration in DNA dilutions (1:1, 1/5 and 1/10) indicated that nine of 22 samples were affected by qPCR inhibition (Table S2), as a higher GC value was obtained in more diluted samples compared to less diluted samples. Subsequent heat treatment of sample DNA (95 °C for 5 min) prior to qPCR reduced this inhibition, as shown by increased HAdV numbers (GC/reaction) detected in the viral concentrates (Fig. 1a, Table S2). When converted to numbers present in the original samples, HAdV concentration was increased by an average of 0.71 log_10_ GC/L (*P* value < 0.05; range −0.9 to 3.04 log_10_ GC/L; Fig. 1b; Table 1). These increases in HAdV concentration due to DNA heat treatment (5 min) are a conservative estimate, since negative samples were assigned a value of 6.25 × 10^3^ GC/L (50% of the assay limit of detection, LOD). Heat treating DNA (5 min) also reduced variability between technical replicates, as evidenced by a mean coefficient of variation (CV%) for heated (5 min) and non-heated DNA of 48% and 78%, respectively. Importantly, in three of 22 of samples from one site, HAdV was not detected at all using unheated DNA, but with heat treated DNA (5 min) 10^4^–10^7^ GC/L HAdV were detected (Table 1). Thus, reporting of false negatives (15% of samples for one site) was reduced by heat treating DNA from the wastewater samples.

**Fig. 1:**
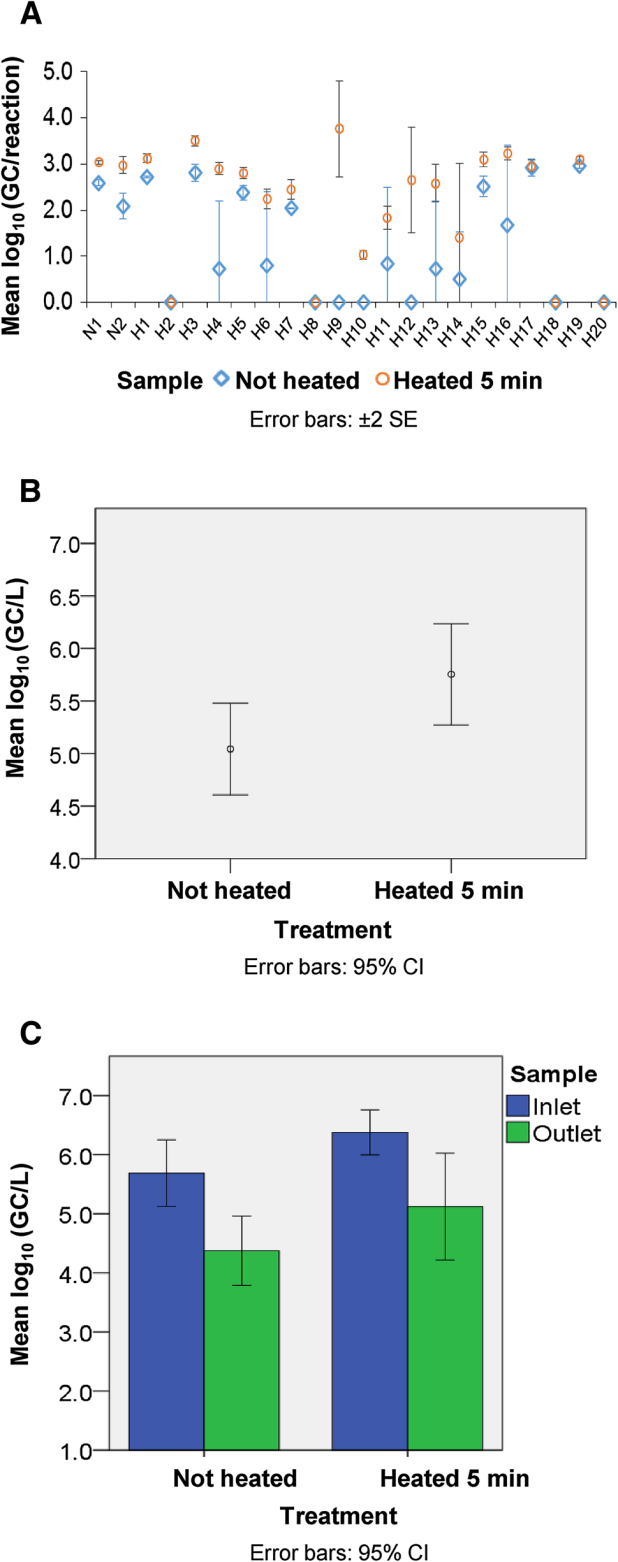
Effect of DNA heat treatment on qPCR estimates of HAdV concentration in wastewater samples. DNA was extracted from viral concentrates of WSP inlet and outlet samples, and DNA that was untreated further (i.e., not heated) was compared to DNA that was heat treated at 95 °C for 5 min before aliquoting into the qPCR reaction. **a** Increases in HAdV concentration (GC/reaction) in viral concentrates due to DNA heat treatment (raw data, mean for triplicate technical replicates). **b** Effect of DNA heat treatment on HAdV GC/L (water sample estimate) for pooled data from 22 samples. **c** Effect of DNA heat treatment on HAdV concentration (GC/L) estimates for inlet and outlet samples from one WSP (*n* = 10 each)

**Table 1:** Effect of DNA heat treatment on qPCR estimates of HAdV concentration in water samples

Sample name	Sample type^a^	Concentration of HAdV (GC/L)^b^			
		DNA not heated		DNA heated 5 min	
N1	I	2.5 ± 0.5 × 10^5^	(1)	6.8 ± 0.5 × 10^5^	(2)
N2	O	8.4 ± 4.8 × 10^4^	(2)	6.2 ± 0.9 × 10^5^	(2)
H1	I	1.4 ± 0.1 × 10^6^	(1)	3.6 ± 0.7 × 10^6^	(1)
H2	O	ND	(1)	ND	(1)
H3	I	1.8 ± 0.6 × 10^6^	(1)	8.8 ± 2.0 × 10^6^	(1)
H4	O	1.5 ± 2.4 × 10^5c^	(2)	2.2 ± 0.5 × 10^6^	(3)
H5	I	6.7 ± 2.0 × 10^5^	(1)	1.8 ± 0.5 × 10^6^	(4)
H6	O	2.3 ± 3.8 × 10^5c^	(2)	5.0 ± 2.0 × 10^5^	(2)
H7	I	3.0 ± 0.1 × 10^5^	(2)	8.0 ± 3.2 × 10^5^	(2)
H8	O	ND	(1)	ND	(1)
H9	I	ND	(1)	1.6 ± 0.3 × 10^7^	(1)
H10	O	ND	(1)	6.7 ± 5.4 × 10^4c^	(2)
H11	I	2.9 ± 4.8 × 10^5c^	(1)	2.0 ± 1.0 × 10^5^	(2)
H12	O	ND	(1)	5.6 ± 9 × 10^5^	(1)
H13	I	1.5 ± 2.4 × 10^5c^	(1)	1.3 ± 1.1 × 10^6^	(1)
H14	O	3.4 ± 4.8 × 10^4c^	(1)	5.5 ± 8.9 × 10^5c^	(1)
H15	I	9.6 ± 3.7 × 10^5^	(1)	3.7 ± 1.1 × 10^6^	(2)
H16	O	8.4 ± 11.0 × 10^5c^	(1)	4.6 ± 1.2 × 10^6^	(2)
H17	I	2.4 ± 0.9 × 10^6^	(1)	2.5 ± 0.6 × 10^6^	(1)
H18	O	ND	(1)	ND	(1)
H19	I	2.5 ± 0.2 × 10^6^	(1)	3.4 ± 0.5 × 10^6^	(1)
H20	O	ND	(1)	ND	(1)

^a^Samples were from the inlet (I) or outlet (O) of WSPs (maturation ponds)

^b^Concentration of HAdV determined by qPCR for water samples using not-heated or 5 min heated DNA. Number in parenthesis indicates diluted/undiluted replicates used for estimation of concentration: 1 = 1:1, 1/5, and 1/10; 2 = 1:1 × 3; 3 = 1/5 × 3; 4 = 1/10 × 3. ND indicates non-detect; NDs were assigned a value of 6.25 × 10^3^ GC/L (50% of the water sample LOD) for calculation of mean differences

^c^Samples where one or two replicates in HAdV qPCR were ND, and other replicates were positive

The effect of DNA heat treatment (5 min) on estimates of pathogen removal by one of the WSPs was also evaluated. DNA heat treatment affected inlet and outlet samples similarly, where HAdV concentration was increased by 0.69 log_10_ GC/L (*P* value > 0.05) and 0.75 log_10_ GC/L (*P* value < 0.05), respectively (Fig. 1c). Due to the comparative increase in both inlet and outlet estimates, the resultant log_10_ reduction value (LRV; an estimate of viral pathogen removal by the WSP) was not affected (LRV unheated = 1.31 log_10_ GC/L, LRV heated 5 min = 1.25 log_10_ GC/L). However, it is important to note that while heat treating DNA did not affect the LRV, ultimately heat treatment did reveal higher estimates of HAdV concentration in the wastewater samples, and also reduced variability between replicates and false negatives.

The overall improved HAdV detection following DNA heat treatment (5 min) is in agreement with results described by Ruano et al. (1992), who showed heat-soaked PCR improved amplification in forensic samples, using three different gene targets. Ruano et al. (1992) also reported that amplification was further improved by heating DNA from forensic samples for 30 min compared to 5 min. However, in the present study, heat treating sample DNA for 30 min (95 °C) resulted in HAdV concentration being reduced by 0.41 log_10_ GC/L compared to unheated DNA (*P* < 0.05, *n* = 16, data not shown), that was potentially related to excessive fragmentation of the small viral genome. Thus, heat treatment of viral DNA from wastewater samples for 30 min was not associated with improved HAdV detection that was observed when the DNA was heat treated for 5 min. Further research might better establish optimal temperature and time combinations for the DNA heat treatment for a given target type. For example, heat treatment at temperatures lower than 95 °C could potentially be effective in destroying inhibitors, while causing less DNA fragmentation, maintaining template integrity and further enhancing amplification. Additional research is also required to better understand the mechanism by which heat treatment can improve qPCR detection. For instance, heat treating viral DNA from wastewater presumably destroyed some inhibitors before they were able to irreversibly modify the DNA polymerase or other reaction components. Alternatively, inhibitors that sequester or entrap the template might have been destroyed, resulting in more even dispersal of the DNA throughout the solution (and thus less variation in replicates). It is also possible that DNA dispersal and DNA polymerase and primer binding, were assisted by fragmentation and denaturation of the template that occurs during heating (Ruano et al. 1992). Further research is also needed to confirm the experimental conditions where DNA heat treatment is effective for improving qPCR results, such as other DNA extraction methods, DNA polymerases/reagents, water and sample types (e.g., less polluted water and sediments), and target types (e.g., RNA viruses and bacteria).

In conclusion, the main advantages of DNA heat treatment, under experimental conditions assessed in this study for wastewater, were improved accuracy of HAdV concentration estimates, reduced variability between replicates and reduced false negatives. Accuracy of quantitative HAdV data is important, since it can significantly impact microbial risk assessment, which estimates the level of health risk associated with pathogen exposure due to re-use of treated wastewater.

## Electronic supplementary material

Below is the link to the electronic supplementary material.

Supplementary material 1 (PDF 546 kb)
