# Supplementary material for: DNA Heat Treatment for Improving qPCR Analysis of Human Adenovirus in Wastewater
Source: Food Environ Virol. 2017 Apr 10;9(3):354–7. doi: 10.1007/s12560-017-9294-4 (PMC5548852; doi:10.1007/s12560-017-9294-4)
Supplement: Supplementary file 1 — Supplementary material 1 (PDF 546 kb) [file 12560_2017_9294_MOESM1_ESM.pdf]

Food and Environmental Virology

DNA heat treatment for improving qPCR analysis of human adenovirus in wastewater.

Emily Rames, Anne Roiko, Helen Stratton and Joanne Macdonald[#].

Genecology Research Centre, School of Science and Engineering, University of the Sunshine Coast, Sippy Downs, Queensland, Australia; jmacdon1@usc.edu.au

Supplementary Methods 1
Water samples and virus concentration 1
DNA extraction and treatment 2
Statistical analysis 3
Supplementary Tables 5
Table S1. Assessment of HAdV qPCR inhibition by spiking reactions with control plasmid. 5
Table S2. Effect of DNA dilution on HAdV qPCR result for heated and unheated DNA. 6
Supplementary References 7

Supplementary Methods

Water samples and virus concentration

Wastewater samples (230 mL – 1L) were collected from the inlet (I) and outlet (O) of the maturation pond, from waste stabilisation pond (WSP) treatment systems as described elsewhere (Sheludchenko et al. 2016). Twenty two samples were assessed in this study: samples N1 and N2 were from a WSP in Northern Territory, Australia; samples H1-H20 were from a WSP in South East Queensland, Australia. Viruses were recovered from the samples and concentrated as described previously (Sheludchenko et al. 2016) as follows. PEG precipitation was performed using a final concentration of 8% PEG 6000, 0.5% v/v 1M $CaCl_2$ and 1% Tween 80 (Li et al. 1998), incubation at 4°C overnight, and centrifugation at 10 000 x *g* for 30 minutes. The pellet was resuspended in 5 mL PBS (pH 7.4) and viruses were eluted by shaking at room temperature for 1 hour with occasional vortexing (Hewitt et al. 2011). The concentrate was extracted with an equal volume of chloroform, to purify viruses from other contaminants (Hewitt et al. 2011; Rodríguez et al. 2012a). The upper aqueous phase was collected and the virus concentrate was stored at -80°C.

DNA extraction and treatment

The virus concentrate from water samples was DNase treated prior to extraction, to remove contaminating bacterial DNA and DNA from non-encapsidated viruses, which ensures the qPCR signal is related to detection of intact viral particles only (Fongaro et al. 2013). For DNase treatment, 20 µL of 10X Turbo DNase buffer and 4 units of Turbo DNAse (Ambion, Life Technologies, Carlsbad, CA, USA) were added to 200 µL of the virus concentrate and incubated at 37°C for 30 min. DNA was extracted from DNase treated virus concentrate (222 µL) using a Qiagen Blood and Tissue extraction kit (Qiagen, Hilden, Germany) according to manufacturer instructions, and resuspended in 100 µL buffer AE.

Quantitative molecular standards

A hexon gene fragment from human adenovirus 41 (Ad41) was used as a molecular standard. A 615bp region (17598 – 18243 bp) of Adenovirus 41 *Tak* type strain (AB330122) was synthesised as a gBlock Gene Fragment (Integrated DNA Technologies, Newark NJ, USA), and cloned into the pCR™4-TOPO® vector (Invitrogen, Carlsbad, CA, USA) according to the manufacturer user guide (ITDNA-gBlock 2015), following dA-tailing of the gBlock with the NEBNext ® dA-Tailing Module (New England Biolabs, Massachusetts, USA). Plasmid DNA was prepared from transformed cells using the QIAprep spin miniprep kit (Qiagen, Valencia, CA, USA). Clones were verified by Sanger sequencing, in both directions using T3 and T7 promoter primers (Australian Genome Research Facility, Brisbane, Australia). The concentration of linearised plasmids (digested with *Spe*I) was determined using a Qubit fluorometer (Invitrogen, Carlsbad, CA, USA). Plasmid concentration was confirmed by diluting to zero copies per reaction, and subsequent absence of signal in HAdV qPCR.

Quantitative PCR

HADV specific qPCR was performed using the primers and Taqman probe described by (Heim et al. 2003) as follows. Each 20 µL qPCR reaction contained 0.5 µM of each forward and reverse primer (AQ2 5'GCCCCAGTGGTCTTACATGCACATC3' and AQ1 5' GCCACGGTGGGGTTTCTAAACTT3'), 0.2 µM probe (AP 5'-FAM-TGCACCAGACCCGGGCTCAGGTACTCCGA-BHQ1-3'), 1X PerfeCTa ToughMix (Quanta Biosciences, Gaithersburg, MD, USA), 0.8 µL 10 mg/mL BSA (Promega, Madison, WI, USA) and 4 µL DNA template (or water in the case of the no template control, NTC). Primers and probes

were synthesised by Macrogen (Seoul, Korea). Cycling conditions were 95°C for 5 min; and 50 cycles of 95°C for 15 s, 55°C for 15 s, 60°C for 60 s (Sidhu et al. 2012). A Rotor-gene Q real-time analyser (Qiagen, Hilden, Germany) was used for all qPCR assays.

A standard curve for each run was generated using a dilution series of 10^5- 10^2 and 20 gene copies (GC)/reaction of Ad41 plasmid in duplicate reactions. Cycle threshold (C_t) and sample GC/reaction, were determined automatically using Rotor-Gene Q Series Software 2.3.1. The limit of detection (LOD) was determined as 20 GC/reaction (C_t = 40). QPCR inhibition was initially assessed for a subset (n=4) of unheated samples, where the qPCR reaction was spiked with 10^3 GC of Ad41 plasmid. Inhibition was considered to be indicated where the subsequent HAdV GC observed was <60% of the expected value (spiked GC + endogenous sample GC). HAdV concentration and qPCR inhibition were further assessed in all samples using 5-fold dilutions of DNA (1:1, 1/5 and 1/10). A given dilution was repeated in triplicate where signal was diluted to below the LOD, inhibition was indicated and/or when results were not consistent in the initial dilution series. For samples where HAdV was not detected, 1/10 diluted DNA was spiked with HAdV control plasmid (10^3 GC/reaction), to determine whether the negative result was due to inhibition.

Statistical analysis

The aim of the statistical analysis was to assess whether there was a significant difference between the mean HAdV concentrations using either heated (5 min) or unheated DNA, for all samples and for inlet and outlet samples analysed separately. The significance of the difference between the mean HAdV concentrations determined using either heated (30 min) or unheated DNA was also assessed for a subset samples (n=16). QPCR data (GC/reaction) were converted to HAdV concentration in the original water sample (GC/L) using the following formula, where where 4 µL DNA is used in each qPCR reaction, 100 µL is the total volume of DNA per extraction and 200 µL of PEG viral concentrate is used for the DNA extraction:

A. GC/4 µL DNA x 25 = GC/100 µL DNA = GC/200 µL PEG
B. GC/200 µL PEG x 25 = GC/5 mL PEG = GC/sample volume (230 mL or 1L)

Non-detects were assigned a value of 6.25 x 10^3 GC/L (half of the sample LOD), such that effects of heating were estimated conservatively (McBride 2005; Carducci and Verani 2013).

The mean HAdV concentration (GC/L) for each sample was determined from triplicate technical replicates (for heated and unheated DNA). Log_{10} transformed data were then used to estimate mean differences for HAdV concentration determined using heated (5 min) and unheated DNA for: 1) all samples (pooled data, n=22); and 2) inlet and outlet samples analysed separately (samples H1-H20, inlet n=10 and outlet n=10). Log_{10} transformed data were also used to estimate mean differences for HAdV concentration determined using heated (30 min) and unheated DNA for a subset of samples (pooled data, n=16). A paired t-test was used to assess significance of differences between the population means (P value < 0.05).

Additionally, the log_{10} reduction value (LRV) in HAdV concentration due to WSP treatment was determined for heated (5 min) compared to unheated DNA (samples H1-H20). LRVs were initially calculated as point estimates, as recommended in the Australian Guidelines for Water Recycling (AGWR 2006):

$$LRV = average(log_{10}(inlet\ GC/L) - average(log_{10}(outlet\ GC/L)).$$

Statistical Package for the Social Sciences (SPSS Version 23, IBM) was used for all statistical analyses.

Supplementary Tables

Table S1. Assessment of HAdV qPCR inhibition by spiking reactions with control plasmid[a].

Sample	Dilution	Heated/ Not Heated[b]	Endogenous qPCR estimate[c]	Spiked with 1000 GC/reaction	
				qPCR estimate[d]	(Expected)
N1	1:1	NH	393	1666	(1393)
N2	1:1	NH	132	601	(1132)
H5	1:1	NH	247	386	(1247)
H8	1:1	NH	0	188	(1000)
H2	1/10	NH	0	829	(1000)
H2	1/10	H	0	934	(1000)
H8	1/10	NH	0	834	(1000)
H8	1/10	H	0	847	(1000)
H9	1/10	NH	0	770	(1000)
H10	1/10	NH	0	862	(1000)
H12	1/10	NH	0	1392	(1000)
H18	1/10	NH	0	649	(1000)
H18	1/10	H	0	564	(1000)
H20	1/10	NH	0	823	(1000)
H20	1/10	H	0	548	(1000)

a. Inhibition was indicated where the observed qPCR estimate was <60% of the expected value (spiked GC + endogenous sample GC)

b. NH = not heated DNA; H = heated DNA (5 min).

c. Endogenous HAdV concentration in a sample was determined from 3 replicates.

d. QPCR reaction was spiked with 10^3 GC of plasmid (n=1).

Table S2. Effect of DNA dilution on HAdV qPCR result for heated and unheated DNA[a].

**Sample**	**Not heated (GC/reaction)**			**Heated 5 min (GC/reaction)**		
	**1:1**	**1/5**	**1/10**	**1:1**	**1/5**	**1/10**
N1	380	70	45	980	331	80
N2	253	39	15	1234	63	0
H1	526	105	49	1085	274	157
H2	0	0	0	12	0	0
H3	886	87	68	4084	558	280
H4	34	0	0	25	0	0
H5	275	33	30	**166**	**54**	**56**
H6	254	84	0	**269**	**415**	**0**
H7	83	19	5	172	27	0
H8	0	0	0	0	0	0
H9	0	0	0	4761	1190	685
H10	14	0	10	205	0	30
H11	**0**	**310**	**0**	175	0	28
H12	0	0	0	**76**	**41**	**589**
H13	**0**	**0**	**158**	**218**	**52**	**98**
H14	33	0	0	**30**	**0**	**58**
H15	**200**	**79**	**46**	2667	108	76
H16	**133**	**0**	**79**	677	33	54
H17	807	252	60	679	227	94

a. HAdV qPCR result (raw data, GC/reaction) produced for individual replicates using undiluted and diluted DNA (1:1, 1/5 and 1/10). Inhibition is indicated in highlighted samples, where a higher GC value was obtained in more diluted samples.

Supplementary References

AGWR (2006). National Guidelines for Water Recycling: Managing Health and Environmental Risks. Environment Protection and Heritage Council, the Natural Resource Management Ministerial Council and the Australian Health Ministers' Conference.

Carducci, A., & Verani, M. (2013). Effects of bacterial, chemical, physical and meteorological variables on virus removal by a wastewater treatment plant. *Food and Environmental Virology, 5*(1), 69-76.

Fongaro, G., Do Nascimento, M. A., Rigotto, C., Ritterbusch, G., da Silva, A. D. A., Esteves, P. A., et al. (2013). Evaluation and molecular characterization of human adenovirus in drinking water supplies: viral integrity and viability assays. *Virology Journal, 10*(1), 1.

Heim, A., Ebnet, C., Harste, G., & Pring-Åkerblom, P. (2003). Rapid and quantitative detection of human adenovirus DNA by real-time PCR. *Journal of Medical Virology, 70*(2), 228-239.

Hewitt, J., Leonard, M., Greening, G. E., & Lewis, G. D. (2011). Influence of wastewater treatment process and the population size on human virus profiles in wastewater. *Water Research, 45*(18), 6267-6276.

ITDNA-gBlock (2015). gBlocks™ Gene Fragments Cloning Protocols. Available online at http://www.idtdna.com/pages/docs/synthetic-biology/gblocks-user-guide.pdf.

Li, J. W., Wang, X. W., Rui, Q. Y., Song, N., Zhang, F. G., Ou, Y. C., et al. (1998). A new and simple method for concentration of enteric viruses from water. *Journal of Virological Methods, 74*(1), 99-108.

McBride, G. B. (2005). *Using statistical methods for water quality management: issues, problems and solutions* (Vol. 19). New Jersey: John Wiley & Sons.

Rodríguez, R. A., Thie, L., Gibbons, C. D., & Sobsey, M. D. (2012a). Reducing the effects of environmental inhibition in quantitative real-time PCR detection of adenovirus and norovirus in recreational seawaters. *Journal of Virological Methods, 181*(1), 43-50.

Sheludchenko, M., Padovan, A., Katouli, M., & Stratton, H. (2016). Removal of Fecal Indicators, Pathogenic Bacteria, Adenovirus, Cryptosporidium and Giardia Oocysts in Waste Stabilization Ponds in Northern and Eastern Australia. *International Journal of Environmental Research and Public Health, 13*(1), 96.

Sidhu, J., Hodgers, L., Ahmed, W., Chong, M., & Toze, S. (2012). Prevalence of human pathogens and indicators in stormwater runoff in Brisbane, Australia. *Water Research, 46*(20), 6652-6660.
